# Towards a Systematic Understanding of How to Institutionally Design Scientific Advisory Committees: A Conceptual Framework and Introduction to a Special Journal Issue

**DOI:** 10.1002/gch2.201800020

**Published:** 2018-09-27

**Authors:** Steven J. Hoffman, Trygve Ottersen, Ali Tejpar, Prativa Baral, Patrick Fafard

**Affiliations:** ^1^ Global Strategy Lab York University University of Ottawa Ottawa Ontario Canada; ^2^ Dahdaleh Institute for Global Health Research Faculty of Health and Osgoode Hall Law School York University Toronto Ontario Canada; ^3^ Division for Health Services Norwegian Institute of Public Health Oslo Norway; ^4^ Graduate School of Public & International Affairs University of Ottawa Ottawa Ontario Canada

**Keywords:** committees, effectiveness of scientific advisory committees, evidence‐based decision‐making, scientific advisory, scientific advisory boards

## Abstract

Scientifically‐derived insights are often held as requirements for defensible policy choices. Scientific advisory committees (SACs) figure prominently in this landscape, often with the promise of bringing scientific evidence to decision‐makers. Yet, there is sparse and scattered knowledge about what institutional features influence the operations and effectiveness of SACs, how these design choices influence subsequent decision‐making, and the lessons learned from their application. The consequences of these knowledge gaps are that SACs may not be functioning as effectively as possible. The articles in this special journal issue of *Global Challenges* bring together insights from experts across several disciplines, all of whom are committed to improving SACs' effectiveness worldwide. The aim of the special issue is to inform future SAC design in order to help maximize the application of high‐quality scientific research for the decisions of policymakers, practitioners, and the public alike. In addition to providing an overview of the special issue and a summary of each article within it, this introductory essay presents a definition of SACs and a conceptual framework for how different institutional features and contextual factors affect three proximal determinants of SACs' effectiveness, namely the *quality* of advice offered, the *relevance* of that advice, and its *legitimacy*.

## 1 Introduction

This special issue on the institutional design of scientific advisory committees (SACs) aims to lay the groundwork for a more systematic understanding of how to institutionally design SACs by examining their effectiveness from the perspectives of multiple global stakeholders across various disciplines. As a whole, the special issue contributes to a better understanding of the complexity of determining the usefulness and the practicality of SACs. Each article offers important lessons to be learned and applied in designing and operating future committees. While certain institutional design features may help improve SAC deliberations, it is worth emphasizing that good evidence alone is an insufficient basis for good policymaking. As such, SAC members must grapple with the fact that science cannot operate in a silo and must take into consideration the larger normative concerns facing policymakers. The scientific evidence advanced in SACs should also reflect local knowledge, as well as the overarching historical and geographic contexts in which advice may be acted upon. SACs can and should play an important role in agenda‐setting. But to be effective, SACs must work in concert with external organizations to alert policymakers to the immediate need to act on time‐sensitive and pressing issues.

## 2 Defining Scientific Advisory Committees

We define a SAC as (a) a group of individuals with relevant expertise (b) that provides advice to decision‐makers (c) predominantly based on research evidence from the natural or social sciences. Instead of “committee,” some use the terms “body” or “panel.” Instead of “scientific advisory,” some use terms such as “expert,” “technical,” or simply “advisory” alone. Some broader understandings of these terms do not completely overlap with SACs as more narrowly defined here. For example, for some other entities, like research ethics boards, the advice given is not always based on evidence from the natural and social sciences. Therefore, for our purposes, a research ethics board is not a SAC.

SAC design varies across multiple dimensions, giving rise to numerous types of these bodies. Some dimensions thought to be important by previous research are shown in **Table** **1**, together with two examples in each dimension.

**Table 1:**
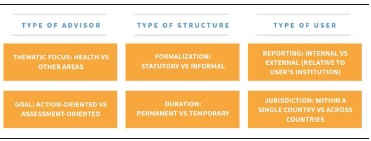
Dimensions of variation across SACs and examples

## 3 What We Already Know

While there is a large and growing literature on the “science” of scientific advice, especially relating to government policymaking,^1^, ^2^ the literature on SACs and the determinants of their effectiveness is sparse and scattered. There is comparatively more literature pertaining to committees that depart from the stated definition of SACs, such as research ethics boards and citizen juries. But at present, there remains a lacuna of rigorous empirical studies on the determinants of SACs' effectiveness.

Instead, most existing work that is relevant is case studies and opinion pieces. This also seems to be the finding of a previous literature review on guideline‐development groups that was undertaken with the goal of improving the design of these bodies.^3^ That previous review found that the existing empirical evidence suggests that committee composition has an impact on the content of their recommendations, but that there is limited research evidence to guide the exact composition of a committee. Against that background, the previous review offered some recommendations for the composition of guideline‐development groups based on the experience of various organizations. The World Health Organization (WHO) has also produced an internal report reviewing the organization's procedures of securing external expert advice, which outlines types and goals of scientific advice, and recommendations based on observations of their own committees.^4^

Among the topics discussed about the mechanics of setting up SACs in the broader research literature pertaining to SACs, several major themes stand out. Such themes include the possibility of collective shirking, where no member does any work on the assumption that others will do it, or groupthink, where members may be unwilling to bring up ideas that go against the majority view.^5^ Another issue is leadership, with some researchers noting the benefits of designating a committee leader,^6^ while others believe that a leader may interfere with other committee members' independence.^7^ The benefits of implementing consensus versus majority decision‐making processes in advisory committees, as well as the degree to which experts interact, have also been closely examined by several studies.^5^, ^8^, ^9^ Researchers have also addressed conflicts of interest and transparency as they relate to SACs.^10^, ^11^, ^12^ The degree to which scientific advice is utilized by policymakers or the public, and the nature of these groups' involvement, have also been the subject of extensive study.^13^, ^14^, ^15^ In addition, there are some useful typologies of SACs;^16^ in particular, a report by Glynn and co‐workers provides a useful overview of national SACs in the European Union.^17^ Finally, while it has not been addressed in many scholarly articles, both the WHO and the UN's Intergovernmental Panel on Climate Change (IPCC) name regional representation as an important consideration when selecting experts for panels.^4^, ^7^, ^9^

## 4 Assessing SAC Effectiveness and its Determinants

The ultimate goal of SACs is commonly seen as informing subsequent decisions with the best available research evidence such that positive impact is maximized and negative (often unintended) consequences are minimized.^18^ In order to achieve this goal there are arguably three proximal determinants for the effectiveness of advice from SACs and indirectly of the effectiveness of SACs themselves: 1) quality; 2) relevance; and 3) legitimacy.^4^, ^19^, ^20^

*Quality* involves the scientific adequacy and accuracy of the committee's advice. *Relevance* relates to the extent to which the committee's advice speaks to decisions to be made.^20^ *Legitimacy* reflects whether the process of generating the committee's advice is respectful of stakeholders' divergent values, unbiased in its conduct, and fair in its treatment of opposing views and interests.^18^

The key question when evaluating the design of SACs is whether the committee is effective. The ultimate indicator of a committee's effectiveness is whether it informed subsequent policymakers' decisions.^18^ Yet, it can also be useful to study the effectiveness of SACs by examining their outputs (i.e., the advice they provide) and outcomes (i.e., behavior change among relevant decision‐makers). The key question for the design, operation, and reform of SACs is what institutional features will make them as effective as possible for their particular context.

**Figure 1:**
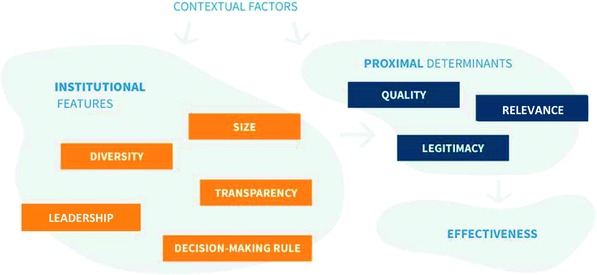
Relationships between institutional features, contextual factors, proximal determinants, and the effectiveness of scientific advisory committees.

Based on the research literature and practical experience with SACs, it is clear that numerous institutional features influence the three proximal determinants of effectiveness. While there are also contextual factors that influence effectiveness, these are often harder to change (see **Figure** **1**).

Most of the institutional features of SACs discussed in the research literature, as well as this special issue, represent intentional design choices that are amendable to change by people seeking to make SACs more effective.^5^, ^6^, ^21^ **Table** **2** lists multiple aspects relating to SACs and contextual factors in their immediate environment that have been proposed as potentially important determinants of their effectiveness.

**Table 2:**
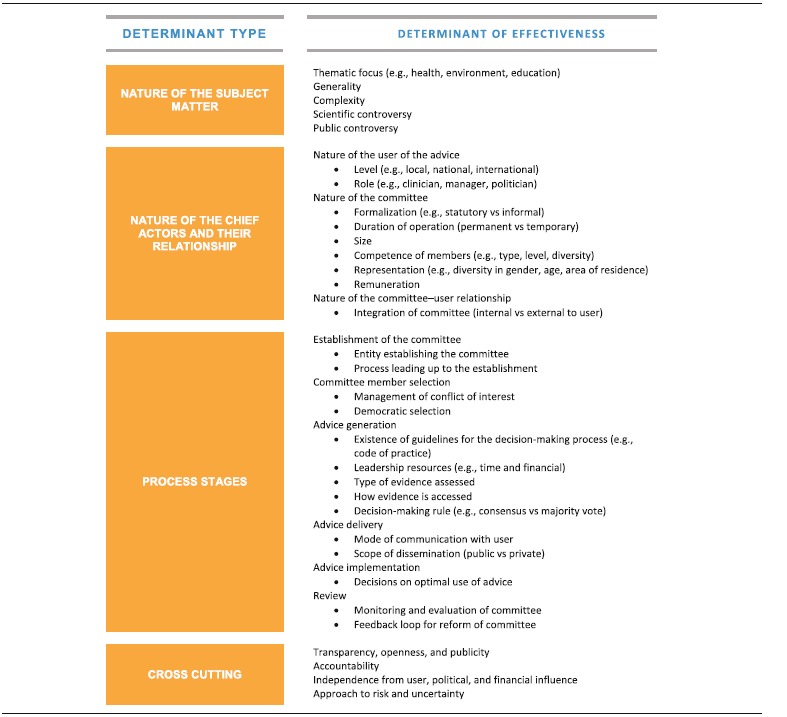
Determinants of effectiveness

The first category of determinants pertains to the subject matter on which the SAC is to provide advice. Determinants include thematic focus, levels of generality, complexity, and controversy. Scientific advice also differs in terms of the extent to which it provides a direct recommendation for action, as opposed to providing a factual assessment or general statements about available scientific knowledge.^15^ For example, some SACs primarily provide forecasts or risk assessments,^17^ including many SACs concerned with environmental risks.^7^ The factors pertaining to the subject matter and nature of advice will, in most situations, function as contextual factors for the design and reform of SACs. However, precisely because of the fact that some SACs will only have a limited impact, it may well be the case that other alternatives, such as commissioning systematic reviews, are sometimes preferable. Understanding how contextual factors influence effectiveness can also help conveners decide whether they can expect any positive effect at all from SACs in any given situation.

The second category pertains to the nature of the chief actors, as well as their relationships. The chief actors in the present context are the SACs and the target users of their advice.

**Figure 2:**
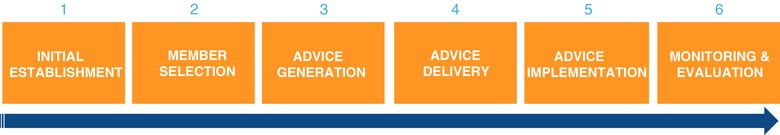
The six stages for the establishment and operation of SACs.

The third category of determinants pertains to the processes in which SACs are directly or indirectly involved, which include at least six stages (**Figure** **2**). The first stage includes the initial establishment of an SAC. The second stage involves selecting its members. The third stage is the SAC's generation of advice, which includes: a) determining what considerations are most important; b) acquiring, assessing, adapting, and applying the available scientific evidence according to these considerations; and c) reflecting on other important information such as perspectives about ethics and equity from other sources such as public consultation. The fourth stage is the SAC's delivery of its advice to the target users, through formal as well as informal channels. The fifth stage is the users' implementation of the advice. SAC members are rarely involved in this latter stage, but this stage is the most important for the effectiveness of the SAC's advice and thereby the effectiveness of the SAC. The sixth stage is the monitoring and evaluation of the SAC's performance and feedback into design and reform efforts.

A fourth category is needed to capture those determinants that cut across all the aforementioned categories. For example, transparency matters when selecting SAC members, holding meetings, releasing work products, drawing conclusions, and disseminating advice. Another cross‐cutting determinant is the approach taken to handle scientific risk and uncertainty. This approach is crucial in assessing evidence as well as communicating advice.

## 5 Overview of the Articles in this Special Issue

The articles in this Special Issue (**Table** **3**) examine the institutional design of a wide range of SACs, with a special emphasis on SACs that provide advice to policy decision‐makers on clinical, health systems, and public health matters at local, national, and global levels.

**Table 3:**
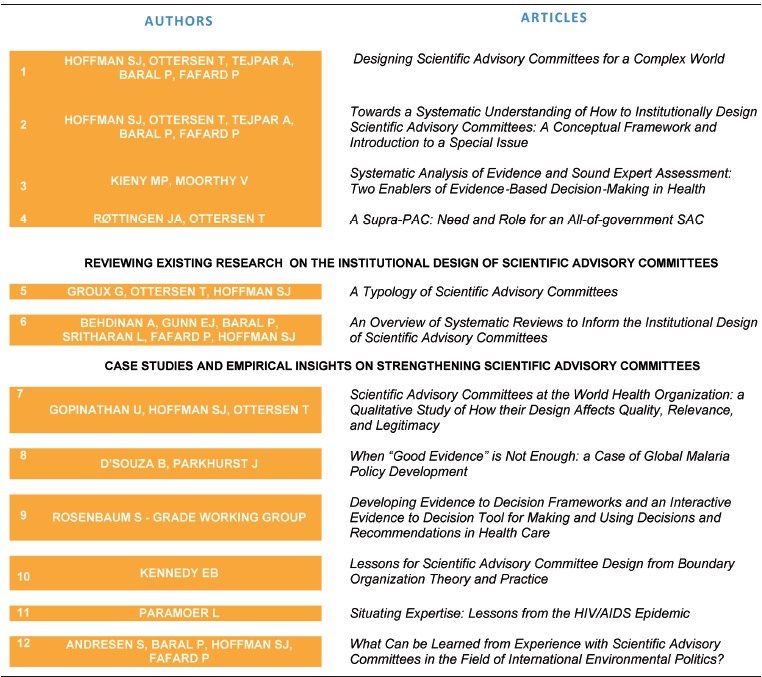
Articles in this special issue on the institutional design of scientific advisory committees

### 5.1 The Need for Scientific Advisory Committees

Following an introductory editorial^22^ and this essay, the special issue features an editorial on the need for “supra‐SACs” or an all‐of‐government approach to SACs.^23^ Rottingen and Ottersen highlight current frameworks used by countries around the world for evidence‐informed policy. They note four questions that governments should consider when establishing SACs. First, should SACs be established for each sector or would an all‐of‐government approach be better? Second, should SACs be involved in substantive policymaking processes or play an oversight role? Third, should SACs contribute solely to generating scientific advice for policy or should they also develop guidelines for the overall use of scientific research? And, fourth, how independent should SACs be from their conveners or sponsors? While Rottingen and Ottersen believe that SACs should be specifically tailored to each national context, they make a case for an all‐of‐government and all‐sector supra‐SAC given the transdisciplinary nature of many societal challenges. They advise that such supra‐SACs should play autonomous oversight and coordinating roles on general policy processes, while still maintaining sufficient links to government to ensure political buy‐in for the use of scientific evidence in policymaking.

An essay was contributed by Kieny and Moorthy, who examine the attributes, membership, and modus operandi of SACs based on lessons they learned as leaders at WHO and specificities of a few selected committees that operate internationally.^24^ More specifically, Kieny and Moorthy use examples of advisory committees in three organizations—WHO, the Wellcome Trust, and the European Commission—to demonstrate structural differences of these committees and the need to strive for a balance of technical expertise, experience, and opinions whatever the mode of constitution may be. They advise that beyond the strength of evidence, advisory committees should consider whether their policy recommendations are suitable, feasible, and acceptable for the specific context. Kieny and Moorthy emphasize the importance of transparency—on membership, on mode of designation and operation, and on the processes used to generate recommendations—in achieving appropriate credibility for the desired outcomes.

### 5.2 Reviewing Existing Research on the Institutional Design of Scientific Advisory Committees

To broaden our understanding of the institutional design of SACs, this series includes two reviews. This first by Groux et al. is a scoping review of the research and gray literature about SACs to outline the current landscape and to develop a typology of SACs along six characteristics.^25^ These include 1) sector, 2) level of operation, 3) permanence, 4) target audience, 5) independence, and 6) nature of advice. Their study illustrates that SACs have become numerous and diverse, operating across all sectors from the local to global levels. In doing so, Groux et al. lay the groundwork to ensure that the design of SACs can be informed by the full range of what options are available and that further empirical studies can be conducted on the impact of variations in SAC characteristics on their effectiveness.

The second review, contributed by Behdinan et al., involved a systematic overview of systematic reviews on the design features of SACs.^26^ The authors searched seven electronic databases, identified 1895 existing reviews, and included six of them based on their inclusion/exclusion criteria. The synthesis of evidence collected by the authors highlight several themes. First, the size of a committee requires a balancing act to ensure that both representation and communication of unique perspectives are met. Second, SACs should be diverse, in terms of members' specialties, demographics, expertise, and views. Third, effective decision‐making processes would benefit from the presence of key procedural determinants of SAC structure, whereby clear protocols delineate responsibilities, group format, and framework. Finally, communication between the SACs and its members is vital to ensure optimal operation. The authors also discuss additional insights around training and group dynamics. Perhaps most importantly, this overview of systematic reviews highlights the current gaps in the research literature on SAC design—of which there are many to be filled by future researchers.

### 5.3 Case Studies and Empirical Insights on Strengthening Scientific Advisory Committees

The series also includes six primary studies that explore various cases and draw insights from SACs in practice. In this study by Gopinathan et al., 35 senior staff members from WHO were interviewed, including department directors and unit coordinators, about the use and effectiveness of SACs at the supranational level.^27^ The interviews yielded five major themes. First, SACs are established to respond to technical needs as well as to serve broader strategic objectives to promote high‐level political messages. Second, ensuring an SAC's independence requires autonomy from the convening institution, the institutions from which its members are recruited, and from the institution receiving the expert advice. Third, designing SACs will often require trade‐offs between quality, relevance, and legitimacy. Fourth, staff supporting SACs must balance between safeguarding decisions from external influence and serving as a broker between SAC members and the external environment. And fifth, SACs must balance the need to involve stakeholders in discussions without compromising the independence and integrity of the scientific process. This study of WHO's SACs provides important lessons on trade‐offs to consider when composing committees.

D'Souza and Parkhurst highlight the importance of ensuring SAC design and functionality actually enable transparent, responsive and credible review of scientific evidence.^28^ D'Souza and Parkhurst undertake a comparative study of two different policy development processes within the WHO Global Malaria Program. Overall, they note that “good evidence” is often not good enough. Or, in other words, that “good evidence” is often insufficient to achieve universal agreement on recommendations. Instead, based on interviews with 29 key informants, they argue that evidence must also be both relevant and usable for policymakers, and reviewed via processes that are accepted as legitimate.

In the next study led by Rosenbaum,^29^ collaborators from the GRADE Working Group developed “Evidence to Decision” frameworks to help groups make well‐informed, systematic, and transparent decisions in health care. The frameworks were developed over a 5‐year period and pilot tested in guideline organizations, including WHO. Using a human‐centered design approach, authors created frameworks that could help SACs explicitly consider the most important factors, and to use scientific evidence together with other kinds of information when making judgments about those factors. They developed frameworks for making clinical recommendations, coverage decisions, and health system or public health recommendations and decisions, as well a free online interactive tool for creating and using frameworks. The “Evidence to Decision” frameworks are a pragmatic approach for bringing a broad range of types of evidence into decision‐making that can also be adapted to other domains. This study reveals the important role technology can play in facilitating structured, well‐informed discussions, and in issuing transparent recommendations and decisions that can more easily be adapted to different contexts of implementation.

Andresen et al. share lessons learned from previous work on the science–policy nexus in the field of international environmental politics.^30^ Specifically, the authors gather lessons learned from using evidence across six international environmental regimes: 1) the International Whaling Commission, 2) the global UN climate regime, 3) the global ozone regime, 4) the North Sea environmental regime, 5) the European acid rain regime, and 6) the biodiversity regime. Above all, the article highlights the important and sobering point that policy is rarely driven by science. SACs must thus balance scientific integrity with political involvement, as researchers must ensure that decision‐makers' needs and concerns are recognized. In the international environmental context, SACs play a crucial role in agenda‐setting but should expect delays before policymakers act. Andresen et al. further acknowledge that scientific warnings about an issue should be supported by other types of actors, and that strong media exposure can help spur immediacy and regime creation.

In the next article, Kennedy explores the use of scientific advice in addressing coastal and ocean issues in California.^31^ In particular, he evaluates the similarities between scientific advising and boundary organizations. He notes that both groups aim to connect science with policy to help inform decision‐making. Moreover, both groups often confront scientific uncertainty, engage in the adjudication of expertise, tend to resolve expertise conflicts through honest brokering, and grapple with balancing science and politics. Specifically, Kennedy considers the roles that both groups played in addressing what to do with decommissioned oil and gas platforms off the California coast. Based on this case study, Kennedy offers important insights on the shared lessons that SACs and boundary organizations can learn from one another. Namely, science plays one part in democratic decision‐making processes, experts facing scientific uncertainty should be transparent in how they evaluate evidence, and expert panels should continuously discuss what it means to develop and provide apolitical political advice. He concludes that bridging the two groups through scientific advising boundary organizations can provide powerful opportunities to cross‐pollinate lessons and practices.

Finally, Paremoer considers three questions related to the work of SACs in the South African health sector.^32^ First, what forms of expertise are typically made invisible? Second, what are the political implications of excluding these forms of expertise? And, third, how can including nonscientific forms of expertise help attain health for all? Paremoer uses the case study of a South African social movement that demonstrated how HIV treatments can be effective in resource‐poor settings, like the township of Khayelitsha. She explores how this movement spurred groups like Medicines Sans Frontiers to prove that HIV‐treatment could be sustained in the Global South, despite concerns that weak public health systems and bad governance were insurmountable obstacles. Paremoer explores how SACs formed after the Khayelitsha case study neglected the evidence that social mobilization and political conscientization were crucial in supporting good trial outcomes. Namely, expert panels failed to consider the importance of social solidarity in promoting sustainable treatment outcomes in resource‐poor settings. Instead, SACs tend to rely on conventional definitions of expertise, such as scientific knowledge. To counter this trend, Paremoer advises that SACs should be hesitant to discount nonscientific knowledge and that they should be transparent about their own political commitments and biases. To aid them, SACs should tackle issues with explicit reference to their relevant history and geography contexts.

## 6 Concluding Thoughts

While much work remains to be done on improving the science of SACs, this special issue has taken steps toward identifying and bridging some existing gaps in the research literature on the institutional design of these bodies. Each article in the special issue contributes to a more rigorous and systematic understanding of how to design, convene, and organize scientific expert committees. This greater understanding, in turn, should improve SACs' effectiveness which will hopefully lead to better policy and program decisions.

The editors of this special issue (S.J.H., T.O., P.F.) would like to thank everyone who made this collection of articles possible, especially our authors, peer‐reviewers, research coordinators, and publishers. We share *Global Challenges*' commitment to open‐access and hope the lessons contained in this special issue will be widely shared and discussed. We hope you enjoy this series of articles and look forward to engaging in further discussions on advancing and improving the institutional design of future SACs.

## Conflict of Interest

The authors declare no conflict of interest.
